# Is the Readmission Rate a Valid Quality Indicator? A Review of the Evidence

**DOI:** 10.1371/journal.pone.0112282

**Published:** 2014-11-07

**Authors:** Claudia Fischer, Hester F. Lingsma, Perla J. Marang-van de Mheen, Dionne S. Kringos, Niek S. Klazinga, Ewout W. Steyerberg

**Affiliations:** 1 Department of Public Health, Centre for Medical Decision Making, Erasmus MC, Rotterdam, the Netherlands; 2 Department of Medical Decision Making, Leiden University Medical Centre, Leiden, the Netherlands; 3 Department of Public Health, Amsterdam Medical Centre, Amsterdam, the Netherlands; Providence VA Medical Center and Brown University, United States of America

## Abstract

**Introduction:**

Hospital readmission rates are increasingly used for both quality improvement and cost control. However, the validity of readmission rates as a measure of quality of hospital care is not evident. We aimed to give an overview of the different methodological aspects in the definition and measurement of readmission rates that need to be considered when interpreting readmission rates as a reflection of quality of care.

**Methods:**

We conducted a systematic literature review, using the bibliographic databases Embase, Medline OvidSP, Web-of-Science, Cochrane central and PubMed for the period of January 2001 to May 2013.

**Results:**

The search resulted in 102 included papers. We found that definition of the context in which readmissions are used as a quality indicator is crucial. This context includes the patient group and the specific aspects of care of which the quality is aimed to be assessed. Methodological flaws like unreliable data and insufficient case-mix correction may confound the comparison of readmission rates between hospitals. Another problem occurs when the basic distinction between planned and unplanned readmissions cannot be made. Finally, the multi-faceted nature of quality of care and the correlation between readmissions and other outcomes limit the indicator's validity.

**Conclusions:**

Although readmission rates are a promising quality indicator, several methodological concerns identified in this study need to be addressed, especially when the indicator is intended for accountability or pay for performance. We recommend investing resources in accurate data registration, improved indicator description, and bundling outcome measures to provide a more complete picture of hospital care.

## Background

Readmissions cause a high burden to healthcare systems and patients. In the US nearly 20% of Medicare patients are readmitted within 30 days after hospital discharge, associated with an estimated annual cost of $17billion [1]. Readmissions are thought to be related to quality of care, for instance due to postoperative complications. As readmissions vary widely across countries, regions and centers, at least part of them might be avoidable [2]–[6]. As a consequence, there is a high interest in the readmission rate as an indicator of quality of hospital care. Nevertheless, the actual way this indicator is used in different countries varies widely.

In the US, since 2009 all-cause hospital readmission rates for pneumonia, congestive heart failure, and acute myocardial infarction are publically reported by the Centers for Medicare and Medicaid Services (CMS) [7]. In 2010, the Patient Protection and Affordable Care Act (ACA) introduced the Hospital Readmissions Reduction Program (HRRP), for cost controlling. The program included financial penalties for hospitals having high readmission rates, which will be extended in the coming years [8]. In the UK, readmission rates for specific diseases have been published since 1998 by the National Centre for Health Outcomes Development (NCHOD) to improve quality [9]. It was found that the crude emergency readmission rate had increased from about 8% in 1998 to about 10% in 2006 [9]. In response, the NHS started a new regulation for reimbursement payments in 2011: hospitals receive no reimbursement for emergency readmissions within 30 days of discharge following an elective admission. All other emergency readmissions are reimbursed for only 25% [10]. Since the year 2006 also the Australian government monitors 28-day readmission rates to gain more insight in quality of care [11].

Readmissions are used for different aims, such as cost control or as balancing measure for length of hospital stay or other outcome measures. However, in recent years the focus has primarily been on using it as an easily available measure of the quality of hospital care. Despite its use by policymakers for both quality improvement and cost control, the validity of readmission rates as a measure of quality of hospital care is not evident [12].

However, in order to consider a quality indicator to evaluate care for external purposes it needs to fulfill certain criteria in regards to its reliability and validity. An indicator needs to show relevance, based on its impact on health, its importance for policy and its susceptibility to being influenced by the health care system. The assessment of an indicator needs to be feasible. The data needed to calculate an indicator need to be available, reliable and need to be seen in relation to the burden of reporting. Further, an indicator needs to show scientific soundness [13]. In the case of the readmission rate, this suggests, that readmissions are determined by quality of hospital care, measured by structures and processes. This implies that we are interested in avoidable readmissions.

We aim to give an overview of the different methodological aspects in the definition and measurement of readmission rates that need to be considered when interpreting readmission rates as a reflection of quality of hospital care for external purposes.

## Methodology

A systematic computerized literature search was applied in the bibliographic databases Embase, Medline OvidSP, Web-of-Science, Cochrane central and PubMed for the period of 1st January 2001 to 27th May 2013.

With the search terms we aimed to cover quality indicators, quality measurement and readmission. This resulted in the following search strategy, which was adapted for the different bibliographic databases: (‘clinical indicator’/de OR ‘performance measurement system’/exp OR ‘quality control procedures’/de OR ‘quality control’/de OR ‘medical audit’/de OR (((qualit* OR perform* OR safet* OR governance) NEAR/3 (indicat* OR measure* OR assessment* OR control* OR marker* OR metric*)) OR ((clinical OR medical) NEAR/3 (indicator* OR audit*))):ab,ti) AND (‘hospital readmission’/de OR (readmiss* OR rehospital* OR ((re OR return) NEAR/3 (hospital* OR admiss*))):ab,ti).

Studies were included when they were written in English, focused on methodological aspects of readmission rates as a quality indicator for hospital care and full texts were available. We included only studies in major disease fields. Hence, studies focusing on rare diseases, just describing readmission rates over time or using readmissions as outcome measures of interventions were excluded.

Of the references identified in the literature search, titles and abstracts were screened and articles that did not meet the inclusion criteria were excluded. The full text of the remaining potentially eligible articles was reviewed to assess whether they should be included. In case of doubt, the article was discussed among the authors and if necessary, an independent researcher was consulted.

We discuss the methodological aspects that emerged from the literature review that are important for the validity of the readmission rates as an indicator of quality of care.

## Results

**Figure 1:**
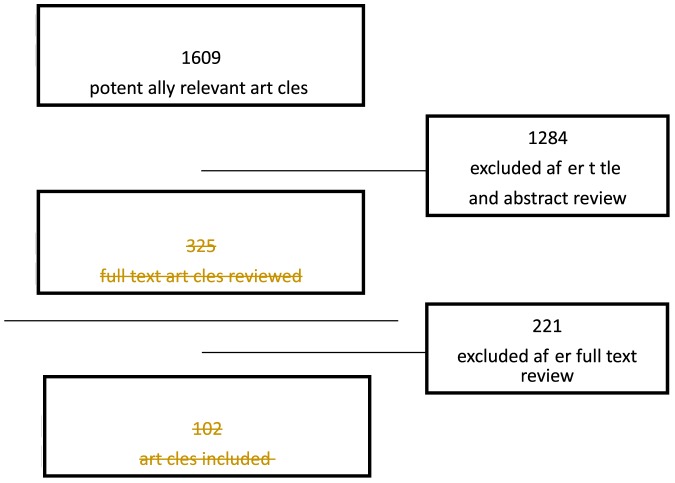
Flow chart of inclusion process.

Our search strategy resulted in 1609 unique references of which titles and abstracts were screened. Based on title and abstract 1189 studies were excluded. Of the remaining 420 articles another 318 were excluded based on full text review (Figure 1). We provide a detailed description of the included studies in the appendix S1, and below we discuss the most important findings.

### The context in which readmission rates are used

Prior to using the readmission rate as a measure of quality of care, the context in which the indicator will be used needs to be clearly defined. The rationale for using readmission rates is one aspect of this context. The readmission rate can be used with the primary aim to improve quality of care or rather for reasons of cost control. Next, specification of the clinical processes of which quality of care is assessed is important. Currently, readmission rates are mostly intended to measure quality of care in hospitals. Which implies that the risk of being readmitted is determined by the quality of care delivered during the hospital stay. Yet, literature shows that the conditions after patients' discharge, like the presence of a social network after discharge [14] as well as patients' capacity for managing their own care, influence the likelihood of being readmitted [15], [16]. As a result, hospitals pay attention to improving transitional care [17]–[24], for instance by patient education to prepare the patient for discharge and to coordinate outpatient follow up [23]. Although such a transition phase may help, the actual post-discharge phase is not really in a hospital's reach anymore. Another example are readmissions in chronic diseases, such as heart failure. These patients are readmitted often because of their comorbidities or because their condition becomes too severe to be treated by the general practitioner, irrespective from the quality of delivered care during their hospital stay [1]. Hence, the quality of care processes captured by readmission rates will often be broader than only in-hospital care [25].

In summary, using readmission rates as a quality measure requires a clear definition of the context, including the rationale of measuring readmissions, the related care processes and the patient groups.

### Methodological aspects

Based on the literature we defined several methodological aspects that need to be considered when using the readmission rates as a quality indicator (table 1). These range from fundamental issues like the definition and the effect of competing outcomes, to more practical issues as the possibility to adjust for case-mix and the data reliability. These issues and their effects will be described in the next paragraphs. In the final paragraph we will focus on studies that have specifically tested the validity of readmission rates as a quality indicator.

**Table 1:** Overview of methodological aspects challenging the validity of readmission rates for benchmarking.

	methodological aspect	problem	potential solution
**Clinical setting**	readmission rates are thought to reflect quality of hospital care (17–24)	care after discharge also influences readmission(14, 25)	clear definition of the indicator, the patient group and the clinical setting(hospital care, integrated care) aimed to measure increase insight in influence of post discharge phase/social factors on readmissions(14)relate readmission to other outcome measures such as mortality, emergency department and observation service use (14) evaluate home health care/nursing home information (15)
**indicator definition**			
Type of readmission	missing distinction between planned/unplanned procedures (2, 26–29)	inclusion of readmissions unrelated to quality of care into the numerator (26) leads to overestimation of the rate of readmission (69)	specify definition of the indicator (27, 28, 31), define disease-specific/emergency readmissions instead of overall readmissions (2) include indication on preventability/avoidability of readmission in definition (2, 28–30)
time window	no consistent definition of the time window in which admission is considered as readmission(28) generally 28–31 day time frame used regardless of patient group/condition(31–33)	although 30 days seems generally sufficient (31, 33), for certain conditions it is a too short time window, while for others it increases the likelihood of including admissions unrelated to index admission(25, 32)	evaluate time frame based on condition under evaluation
**effect of competing outcomes**			
association with (in-hospital) mortality	a group of patients who receive poor quality of care are not readmitted, because they die or recover nevertheless (31, 34, 35)	not excluding patient who died from the denominator leads to a potential underestimation of rate of qoc related readmission	exclude patients who died during hospital stay from denominator link hospital data with death statistics, exclude patients from denominator who die outside hospitalrelate the readmissions with mortality rate in order to understand total hospital performance (36, 37)
association with length of in-hospital mortality	a decreased length of hospital stay increases readmissions (38, 39)	the exact mechanism with readmission is inconclusive (24, 39, 40, 42–50)	further research to understand the mechanism between length of stay and readmission
**case mix adjustment using administrative data vs. clinical data**	no consensus on which patient characteristics affect readmission likelihood(27, 52) two high risk groups defined: the sickest and the poorest (2, 51, 53, 54)	these factors are not standard variables in risk prediction models as often not available in administrative databases(36) current risk prediction models perform moderately (40)(39, 55–57)	apply proper case-mix adjustment for patient characteristics including socioeconomic status and disease severity(39, 51)further research on risk prediction models including linkage of primary care data and socioeconomic information
**data reliability**			
missing readmissions to other institutions	patients are readmitted to institutions other than index hospital (25, 35)	patients cannot always be followed between centers; only readmissions to same institutions are measured assessing “same hospital” readmissions, might be underestimation of the real number of readmissions(25)	further research on the proportion of patients readmitted to other hospitals than index hospitalunique patient information to follow patients between centers
coding	coding practice influences the validity readmission rates(30, 58, 77–84) no conclusion on how to register readmissions potentially related to qoc in reliable way (2, 59–61)	missing distinction between planned/unplanned procedures leads to overestimation of real readmission variation in coding leads to biased comparison between hospitals	increase investment in performance measurement systems(16) research on data reliability(28)standardized data registry (electronic data systems)(16, 62)engagement of the provider in measurement, analysis and interpretation of the indicator(16, 64)
completeness and accuracy of data source	reliable data collection systems are lacking(38) Readmissions are mainly calculated based on administrative data(16, 63) administrative data suffer from inaccuracy, like non-exact/incomplete registration of variables not relevant for financial concerns(39, 40, 64–69)	incomplete registration may lead to over/underestimation of real readmission inaccurate indication of readmissions related to qoc may lead to overestimation of readmissions (64, 65)	aim for minimum data set with complete registrationregistration of unique patient identifying information to enhance possibility for linking data(such as pharmacy data)(70) enhancing linkage opportunities increases possibility for better case-mix adjustment
validity of readmission rates as a quality measure	no gold standard on how to assess qoc in the literature huge variation in conclusions in regard to the validity of the readmission indicator (71–113)	potentially invalid conclusions on qoc	above described methodological conditions need to be taken into account when further investigating readmissions as a quality indicatoradditional data gathering for further investigation of outlier hospitals(93)

### Indicator definition

#### Type of readmission

The definition of readmissions determines the number of readmissions that will be counted (numerator). Planned procedures, such as staged operations, are readmissions that are not determined by quality of care and therefore should not be included in the numerator of the quality indicator [26], [27]. However, this basic distinction is not always made [28]. Hence, capture quality of care related readmissions requires a more specific definition (such as disease specific or emergency readmissions) rather than all-cause readmissions. [29].

A frequently suggested alternative is to count unplanned readmission rates. However, not all unplanned readmissions are a result of poor quality of care as certain complications cannot be avoided. Research has shown that just about 25% of all readmissions are avoidable/preventable. Therefore, ideally, the addition on whether a readmission was avoidable/preventable. Although high variation in overall readmission rate can be observed, this is not the case for the rate of preventable readmissions [2], [29]. Therefore, ideally it is defined, whether a readmission was avoidable/preventable (through proper care delivery) [28], [30] but the judgment on the preventability of a readmission remains subjective [2].

#### Time window

The time window after the index admission in which admissions are regarded as readmissions is not consistently defined in the literature. The indicator is generally calculated on basis of readmissions within one month (28 days UK, 31 days USA) regardless of the patient group and condition [28], [31]–[33]. When choosing a time window, it needs to be considered that a too short time window might miss related readmissions while a large one increases the likelihood of included admissions unrelated to the index admission. For example, in cancer surgery a longer time frame would allow to provide a better overview of actual costs, but it would also include readmissions due to disease progression instead of poor quality of surgery [25]. Clearly, the type of disease the patient was originally treated for is largely influencing the optimal timeframe [32]. Therefore the timeframe for readmissions should be defined per disease.

### The effect of competing outcomes

#### Association with (in-hospital) mortality

Mortality can be seen as a competing endpoint for readmissions: patients who die will not be readmitted [34], [35]. Therefore patients who died during their hospital stay need to be excluded from the denominator of the readmission rate. Further, hospitals with high 30-day in-hospital mortality rates are not necessarily outliers on the readmission rate as well [36]. Research showed that the link between high readmission rates and mortality rates on hospital level is limited. A “modest” inverse relationship was merely found for heart failure patients, and no relation could be observed for pneumonia and acute myocardial infarction, suggesting that the two indicators measure different aspects of quality of care, which are not strongly related [37]. Therefore different outcome measures, such as the readmission rate and the mortality rate should be brought in relation with each other to gain insight in total hospital performance [36], [37].

#### Association with length of in hospital stay

Length of stay is generally decreasing, partly because of efficiency gaining interventions, such as a “just-in-time bed availability system” to increase the bed turnover ratio [38], [39]. Research suggests a link between length of stay and the risk of being readmitted [39]–[45]. For each day shorter in hospital, a 6% increase in likelihood of readmission was found [40]. Other studies fail to confirm this link [24], [42], [46]–[50], which might be due to inappropriate adjustment for disease severity [41], [51].

### Case mix adjustment

The likelihood that a patient is readmitted is not only affected by quality of care but also by characteristics of the patient. Between-hospital differences in readmission rates may be caused by differences in patient population and therefore readmission rates need to be adjusted for patient characteristics. Although many case-mix adjustment models for readmissions have been developed, there is little consensus on which patient characteristics affect the likelihood of a readmission [27], [52]. Numerous studies, varying in their methodology, geographical characteristics, patient groups and considered variables, find different factors that increase the risk of re-admission. In general, two patient groups seem to be at a high risk of being readmitted: the sickest and poorest patients [2], [20], [51], [53], [54]. However, these factors are often not included as standard variables in case-mix adjustment models, as these models are often based on administrative data and therefore miss detailed clinical information.

In a review that evaluated 30 validated readmission risk prediction models, the authors concluded that most models had poor predictive ability. Almost all studies had c-statistics less than 0.70 [55], possibly due to missing demographic or clinical variables. In a more recent paper, the prediction model reached a higher predictive ability (c-statistic = 0.80) [41]. The authors concluded however that information on demographics, SES, prior utilization and diagnosis still had restricted predictive power [41]. Thus, current research provides limited guidance on which variables should be included in models to adjust for case-mix [41], [55]–[57].

### Data reliability

#### Missing readmissions to other institutions

Not all patients are readmitted to the same center where they had their index admission. This is mainly due to the centralization of complex operations in tertiary centers, such as in oncology [25]. When patients unexpectedly develop complications and are readmitted in their local center, they are not captured when only readmissions to the “same hospital” are counted [25]. Missing these patients leads to an underestimation of the true overall readmission rate.

#### Coding

The coding practice within a hospital has an essential impact on the validity of readmission rate as a quality indicator [58]. The way a “planned” procedure is defined is crucial for the comparability between hospitals. Ideally a planned readmission is coded in the registration system, for example, with an additional coding element “staged” at the index admission, which would indicate that a follow-up procedure is planned [59].

Urgent readmissions are sometimes considered as a potential proxy for the relatively subjective ‘avoidable readmissions’, as these are coded, for example an admission through the ER. Although low urgent readmission rates showed to be related to low avoidable readmission rates [60] it was shown that the “avoidability” of urgent readmissions also significantly varied by the time from discharge, with early readmissions being more likely to be avoidable [2], [61].

Other causes for biased comparisons between hospitals are the different and unspecific definitions of the type of readmissions assessed, and variation in coding between hospitals. It is essential who is in charge of the coding process. For example administrative staff at the department or hospital level, the treating clinician, or specialized data coders. The variation in coding practice may affect both the readmission rates and the case-mix variables.

#### Completeness and accuracy of data source

Electronic health records and health information exchange networks result in more accurate and complete clinical data [62]. The major information source to calculate the readmission rate is administrative data. The advantage of administrative data is that this data is standard available and patient journeys can be followed (within hospitals) [63]. Nevertheless, one major limitation of administrative data is the data inaccuracy [64], which includes the non-exact or incomplete registration of variables that are not relevant for financial concerns [38], [40], [41]. Research showed that to a certain degree administrative data captures similar information compared to medical records, for example on all-cause readmissions [65]–[68]. However more specific information, like the identification of unplanned readmissions or index procedure related readmissions, showed to be more difficult to extract [66], [69]. An accurate indication of whether a readmission is a part of treatment or due to a cancelled procedure and not a readmission related to a quality of care problem, would enhance the reliability of the data source [64], [65].

The case-mix adjustment variables that have been investigated so far are most often present in administrative databases. However, clinical information such as disease severity is often lacking limiting case-mix adjustment possibilities. The addition of a unique patient identifier across different databases would enhance the possibility for linking data, such as pharmacy data [70] or clinical data. This would largely improve the possibilities for more precise definitions of readmissions and better case-mix adjustment.

### Validity of readmission rates as a quality measure

No gold standard exists on how to assess quality of care. Usually different hospital structures and processes and their relation with patient outcomes are measured. The different definitions and proxies used in studies to quantify quality of hospital care influence whether an association between the readmission rate and ‘quality’ is found. For example, we found studies that relate readmissions to hospital volume, but neither can be regarded as a ‘gold standard’ of hospital quality.

Furthermore, the methodological aspects we discussed have a potential influence on the validity of the readmission rates as a quality indicator. These may contribute to the huge variation in conclusions with regard to the validity of readmission rates found in the literature. Different studies in different patient groups and conditions come to the conclusion that lower quality of hospital care is linked to a higher number of readmissions [71]–[94]. Especially safety-related events (such as postoperative complications) show a relation with readmissions [71], [95]. Rosen and colleagues, who evaluated the correlation between patient safety indicators and readmissions, showed that patients who experienced a patient safety event had an increased risk of readmission [71]. Nevertheless, there are also studies that are inconclusive [96]–[101], show an inverse relationship [102], [103] or no relationship at all between readmission rate and in-hospital quality of care [98], [104]–[113]. Analysis of additionally collected data could help to gain insight into outlier hospitals in order to understand driving mechanisms behind high readmission rates [93].

## Discussion

This review aimed to summarize the methodological aspects that need to be considered when using the readmission rate as a measure for quality of hospital care for external purposes. We found that the validity of readmission rates as a quality indicator is influenced by the clinical process that is assessed, the indicator definition, the extend of case-mix correction, the effect of competing outcomes and the data reliability. Ignoring or poorly handling these aspects may lead to a biased estimation of the overall readmission rate and a biased comparison of readmission rates between hospitals. As a result of variance in handling these methodological threats, studies on the validity of readmission rates as a quality indicator reach conflicting conclusions. We conclude that given the limitations of readmission rates, they need to be used with caution as a measure of in-hospital quality, even more when used as a tool for a pay for performance scheme.

Some of the discussed factors concerning the readmission rate could in principal be improved by investing resources in accurate data registry and refinements of indicator description. For instance, by using unique patient identifiers to follow patients across centers. That would help to avoid missing readmissions to other institutions. Another option would be to flag planned admissions, which are a part of the treatment plan or due to cancelled procedures, to measure just the quality of care related readmissions.

Other problems, such as the competing endpoint “mortality” are more complex. Patients who died in hospital need to be excluded from the patient group forming the denominator to calculate the readmission rate, as they are not at risk any more to be readmitted. These deaths are captured in the mortality rate. Therefore it is essential to combine outcome in order to provide a more complete picture of the quality of hospital care.

Nevertheless there are theoretical considerations whether a readmission is an indication of bad quality of care. First, a readmission is obviously a more positive outcome than dying. Secondly, if there is for example a chance of six percent that a complication occurs after discharge, it would mean that 100 patients need to be admitted longer, to avoid a complication in six patients [114]. It can be questioned whether by a longer length of hospital stay a complication really can be avoided or only detected at an earlier stage. It is also possible to inform the patient on the risk of developing a complication and decide together how to continue. Furthermore, it needs to be taken into account that readmissions are not always solely determined by quality of hospital care. For certain diseases, like heart failure, the patient's condition is the major driver behind repeated admissions. Patients with low socioeconomic status, elderly and patients with co-morbidities are at high risk of getting readmitted. Therefore case-mix adjustment is essential. Furthermore, the role of facilities outside the hospital and after the 30day time window, like community services, need to be involved in the conceptual framework of readmissions. When aiming to improve quality of care (in and outpatient) increased integration and cooperation between primary and secondary care is needed.

The literature study revealed inconclusive results for some methodological aspects, such as the relation with length of stay, or patient characteristics. The studies we assessed investigated different patient populations and often were based on hospital administrative data. A recent high quality study which was not included in our review investigated surgical readmissions of 479,471 patients from 3004 hospitals. The authors found that higher surgical volume was significantly related with lower composite readmission rates (upper volume quartile 12.7% vs. lower volume quartile 16.8% P<0.0001), and hospitals with the lowest surgical mortality rates had significantly lower readmission rates (lower mortality quartile 13.3% vs. upper mortality quartile 14.2% P<0.0001). But high adherence to surgical process measures was only marginally linked with lower readmission rates (highest quartile vs. lowest quartile, 13.1% vs. 13.6%; P = 0.02), showing that it is still unclear whether low readmission rates are the result of good quality [115].

Furthermore, the risk of getting readmitted is also varying between patient groups and conditions. This supports the idea that outcome measures, like the readmission rate, are not a one size fits all measure. Even if quality of hospital care and the transition phase can potentially be improved, readmissions might be a more applicable measure for certain diseases than for others. For chronic diseases, where planned admissions are part of treatment strategies, readmissions are a less suitable performance measure. At least not until generally used data systems can identify planned admissions with high certainty. It requires clinical knowledge to determine whether (avoidable) readmissions may theoretically represent poor quality of care for specific diseases. Consequently more research is needed to build reliable algorithms to identify avoidable readmissions.

In sum, avoidable readmissions are of high relevance, as they are an adverse event to patients and family and are a high financial burden for healthcare systems. The assessment of the indicator shows difficulties, as the indicator definition is often not explicit enough to identify readmissions related to quality of care (avoidable readmissions). The data used to calculate the indicator is mainly administrative data, which generally includes incomplete and inaccurate data elements and lacks clinical information. Furthermore, in many countries readmissions to other institutions cannot be followed. Readmission rates are influenced also by other factors than quality of hospital care, which include length of stay, (in-hospital) mortality and patient characteristics. The magnitude of influence is partly not know as data is missing to investigate the association (e.g. no post discharge mortality, no clinical characteristics). Further, the scientific evidence of the indicator is limited, as existing research shows conflicting results with regard to the influence of quality of hospital care on the readmission rate (see Appendix S1). This, however, could be related to the prior mentioned methodological aspects that are variously.

Using outcome measures externally to measure and compare hospital performance has consequences. When financial consequences are linked to the outcome, unintended effects could occur. For example, hospitals may try to reduce their readmission to escape the penalty of exceeding the readmission rate by lowering admissions, moving readmissions after the 30-day window, or risk-avoidance in regards to high risk groups. These gaming efforts might reduce the focus on the actual intention: improving quality of hospital care.

A measure used for external purposes should be underpinned with solid evidence for its validity. However, the link between readmissions and the quality of hospital care seems not to be fully explained yet. Still, this does not imply that there is no room for improvement for hospitals in their readmission rate and the indicator could not be useful for internal use. Research should continue to gain insight in the driving mechanisms behind readmissions for the different conditions to improve our understanding how the readmission rate is a part of the quality of hospital care picture. In addition, the readmission rate needs to be brought into relation with other outcome indicators, and hence considered as part of a bundle, to understand all aspects of hospital performance [36].

The methodological aspects we identified need to be considered when using readmission rates as quality indicator. The use of readmission rates for external quality purposes, such as for pay for performance requires strict methodological criteria to avoid confounding. At its current state the rate of readmission does not fulfill the methodological requirements of a reliable and valid indicator. Therefore the indicator should not be used for external purposes. As this is nevertheless currently happening, readmission rates should be interpreted with great caution.

## Supporting Information

Checklist S1 **PRISMA checklist.**
(ZIP)

Appendix S1 **Descriptive information of included studies.**
(EPS)
