# Correction: Is the Readmission Rate a Valid Quality Indicator? A Review of the Evidence

**DOI:** 10.1371/journal.pone.0118968

**Published:** 2015-02-27

**Authors:** The PLOS ONE Staff

There is an error in the first sentence of the Results paragraph of the Abstract. The correct sentence is: The search resulted in 101 included papers.

There is an error in the third sentence of the first paragraph of the Results. The correct sentence is: Of the remaining 420 articles another 319 were excluded based on full text review (Fig. 1).

There is an error in Fig. 1. The authors have provided a corrected figure below.

**Fig 1:**
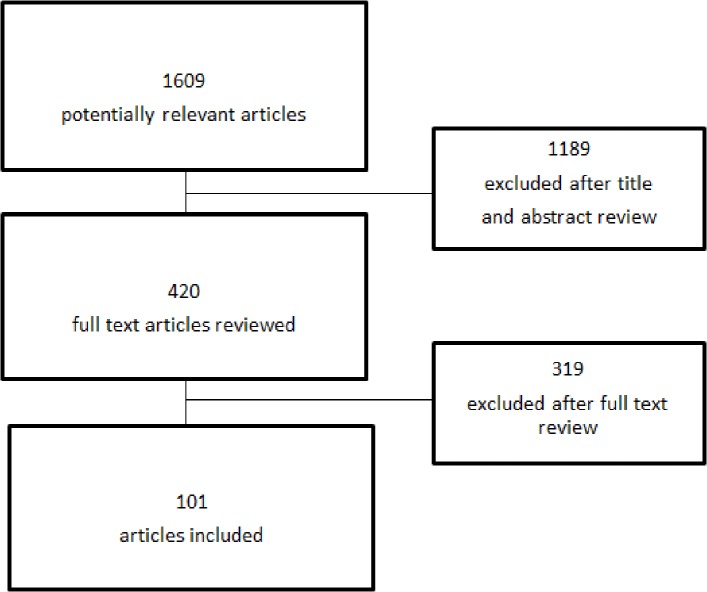
Flow chart of inclusion process.
